# Elevated Serum Chromogranin-A and Characteristic Duodenal Enteroendocrine Cell Distribution in Pancreatic Fibrosis and Chronic Pancreatitis Compared with Other Dyspeptic Disorders: A Case Series Study

**DOI:** 10.3390/diagnostics15192428

**Published:** 2025-09-23

**Authors:** Chung-Tsui Huang, Yao-Jen Liang

**Affiliations:** 1 Division of Gastroenterology, Department of Internal Medicine, Far-Eastern Memorial Hospital, No. 21, Sec. 2, Nanya S. Rd., New Taipei City 220, Taiwan; 950286169@mail.femh.org.tw; 2 Graduate Institute of Applied Science and Engineering, College of Science and Engineering, Fu Jen Catholic University, No. 510, Zhongzheng Rd., New Taipei City 24205, Taiwan

**Keywords:** duodenum, enteroendocrine cell, pancreatic fibrosis, chronic pancreatitis, distribution

## Abstract

**Background**: Prior research has reported increased expression of duodenal chromogranin-A (CgA), secreted by enteroendocrine cells (EECs), in association with pancreatic fibrosis. However, it remains unknown whether serum CgA levels are also elevated, and whether there is a different distribution pattern of EECs in pancreatic fibrosis and other dyspeptic causes. Aims: This study had three main objectives. First, to compare the serum CgA level between patients with pancreatic fibrosis and those with other dyspeptic conditions. Second, to analyze the distribution pattern of duodenal EECs. Third, to evaluate whether biopsy results varied depending on the specific location within the duodenum. Serum CgA levels were categorized into low and high groups based on a cutoff value of 50 ng/mL. **Methods**: This cross-sectional prospective case series included 15 patients, with 4 patients in the low CgA group and 11 in the high CgA group. Each participant underwent a serum CgA test, transabdominal ultrasonography, pancreatic elastography, and upper gastrointestinal endoscopy. During endoscopy, a single gastric biopsy and three duodenal biopsies from different locations were obtained. **Results**: Patients in the high CgA group were generally older (52–68 years) than those in the low CgA group (37–55 years), with a statistically significant difference (*p* < 0.01). The high CgA group exhibited a clustered and centralized pattern of EECs, whereas the low CgA group showed a more discrete pattern with fewer EECs (*p* < 0.01). All duodenal ulcer cases were found in the low CgA group, while three cases of pancreatic fibrosis and one case of chronic pancreatitis were identified in the high CgA group. In the high CgA group, five cases of functional dyspepsia showed a band-like EEC distribution pattern, whereas cases with pancreatic fibrosis demonstrated a more uniformly scattered EEC distribution (*p* < 0.01). Consistency among intra-individual duodenal biopsy results was high across different biopsy sites. **Conclusions**: Elevated serum CgA (>50 ng/mL) and specific duodenal EEC distribution patterns may serve as potential diagnostic indicators for pancreatic fibrosis or chronic pancreatitis. These characteristics could help differentiate these conditions from functional dyspepsia.

## 1. Introduction

Uninvestigated dyspepsia represents a prevalent concern in digestive medicine, with a global prevalence reaching up to 17% [1]. Its etiologies are generally categorized as functional or organic dyspepsia [2]. Non-malignant organic causes include *Helicobacter pylori* infection, peptic ulcer disease, gallstones, pancreatic fibrosis or early chronic pancreatitis, and chronic pancreatitis. Endoscopy is commonly employed as a definitive diagnostic tool to evaluate dyspeptic etiologies such as peptic ulcers or *Helicobacter pylori* infection [2]. Additionally, transabdominal sonography with elastography has proven valuable for diagnosing gallstones and pancreatic diseases, particularly pancreatic fibrosis or early chronic pancreatitis. Among these etiologies, early chronic pancreatitis is a relatively novel disease concept and remains less extensively studied [3,4,5]. In this study, the term “early chronic pancreatitis” is substituted with “pancreatic fibrosis” due to the evolving nature of its diagnostic criteria. Notably, pancreatic fibrosis is recognized as the primary pathological basis of early chronic pancreatitis. Therefore, the use of “pancreatic fibrosis” is deemed more appropriate, reflecting its acceptance as a non-histological diagnosis supported by imaging evidence and established clinical understanding [6].

Pancreatic fibrosis is a significant and emerging contributor to organic dyspepsia [7]. Transabdominal elastography offers a less invasive and more cost-effective imaging modality; however, its sensitivity in identifying late-stage chronic pancreatitis is approximately 89% [8], and it is likely lower in detecting pancreatic fibrosis [9]. Consequently, there is a critical need to develop specific diagnostic markers for pancreatic fibrosis, which would ultimately enhance the early detection and diagnostic accuracy of early-stage chronic pancreatitis.

In medical practice, fundamental diagnostic procedures such as transabdominal ultrasonography and upper gastrointestinal endoscopy are routinely employed to evaluate structural abnormalities of the digestive system in patients presenting with dyspepsia. However, with the advent of precision medicine, there is an increasing emphasis on cellular analysis. Specialized gastrointestinal cells responsible for gut hormone secretion have become a significant focus of research due to their systemic effects. Enteroendocrine cells (EECs) serve as a critical link between the pancreas and gastrointestinal tract. They regulate digestive processes by secreting gut hormones, which function as chemical messengers that travel through the bloodstream to influence target organs such as the pancreas [10]. EECs are distributed throughout the stomach, duodenum, pancreas, and other components of the gastrointestinal system. Chromogranin-A (CgA), a granin family neuroendocrine protein, is commonly expressed in neuroendocrine cells and is recognized as a marker for EECs [11]. In this study, CgA-stained cells are utilized as surrogates for EECs.

Previous studies have reported that duodenal mucosal CgA expression is elevated in patients with pancreatic fibrosis compared to those with functional dyspepsia [12]. This observation has led to the hypothesis that CgA may serve as a valuable marker for the complementary diagnosis of pancreatic fibrosis. To validate this hypothesis, three key questions must be addressed. First, does serum CgA correlate with duodenal mucosa CgA expression? A positive association would suggest that serum CgA could function as a serological marker for pancreatic fibrosis. Second, is there a correlation between the histological distribution pattern of duodenal mucosal EECs and duodenal mucosa CgA expression? If correlated, duodenal mucosal biopsy for histological evaluation of EECs may provide pathological support for diagnosing pancreatic fibrosis. The rationale is that EECs may undergo dynamic changes in response to injury affecting the gut and other organs [13]. Third, what are the critical considerations regarding the biopsy procedure for accurately assessing duodenal EECs, including the optimal biopsy sites and the number of specimens required? Regarding the second question, the pattern of duodenal mucosal EECs can be analyzed based on cell quantity or distribution. This study focuses on cell distribution patterns, as recent research has demonstrated that specific patterns of cell distribution can influence disease presentation and outcomes [14,15].

This study has three primary objectives. The first objective is to compare serum CgA levels between individuals with pancreatic fibrosis or chronic pancreatitis and those with other dyspeptic conditions. Identification of significant differences may suggest that serum CgA could serve as a diagnostic marker for pancreatic fibrosis. The second objective involves analysis and comparing the distribution patterns of duodenal mucosa EECs among patients with pancreatic fibrosis and other disease groups. The third objective is to evaluate whether biopsy results differ in the same individual. For the first objective, given the broad reference range for serum CgA (1–101 ng/mL), this study arbitrarily categorized serum CgA into high and low groups using a 50 ng/mL cutoff. This classification was predicated on the hypothesis that high and low serum CgA levels are significantly associated with different non-malignant dyspeptic etiologies. Overall, the purpose of these study objectives was to find a diagnostic approach to differential diagnosis of pancreatic fibrosis, which could be applied to clinical use.

## 2. Methods

This study received approval from the Institutional Review Board of Far-Eastern Memorial Hospital (FEMH) under case number 110194-F. It was designed as a clinically prospective study conducted according to protocol. Informed consent was obtained from participants prior to the start of the study. Procedures and management adhered to the ethical standards outlined in the Declaration of Helsinki. Data was collected from clinics specializing in digestive medicine at FEMH.

The study included patients aged 35 to 70 years with symptoms of dyspepsia. These participants were surveyed and enrolled at digestive clinic of FEMH. Exclusion criteria comprised individuals with surgically altered anatomy, increased risk for endoscopy, suspected or untreated malignancy, and pregnancy. Each participant underwent three assessments: laboratory evaluation of chromogranin A (CgA), lipase, and white blood cell counts; upper gastrointestinal endoscopy (Olympus Co., Tokyo, Japan); and trans-abdominal sonography evaluating the liver, gallbladder, bilateral kidneys, spleen, and pancreas. Acoustic radiation force impulse elastography via trans-abdominal sonography was performed on the pancreas in all cases using the Siemens Acuson S2000 Ultrasound System (Munich, Germany). During upper gastrointestinal endoscopy procedures, detailed examination of the esophagus, stomach, and duodenum were performed. Gastric mucosal biopsy specimen was obtained from the gastric antrum for *Helicobacter pylori* and atrophic gastritis-related intestinal metaplasia surveillance. Duodenal mucosal biopsies were performed at three distinct locations on the anterior wall of the first part of the duodenum—upper, middle, and lower positions—to analyze EEC distribution patterns. The rationale for collecting biopsies from multiple sites was to compare potential variation in EEC distribution within the same individual.

The demographic data of enrolled patients comprised age, gender, history of cigarette smoking and alcohol consumption, non-steroidal anti-inflammatory drug usage, comorbidities, prior abdominal surgeries, as well as the characteristics, duration, and course of dyspeptic symptoms. Laboratory parameters included white blood cell count and serological assessments of lipase and CgA. Gastric and duodenal biopsy specimens were submitted to pathology departments and evaluated by physicians who were blinded to patient information. Gastric mucosa was assessed for the presence of *Helicobacter pylori*. Three biopsies from different locations of the duodenal bulb were stained for CgA. Pancreatic acoustic radiation force impulse elastography measurements were performed five times, with the median value recorded in meters per second (m/s) [16]; the region of interest was selected at the pancreatic body to minimize interference from bowel gas. Pancreatic fibrosis was defined by acoustic radiation force impulse velocities greater than 1.4 m/s or by imaging evidence of fibrosis, whereas late-stage chronic pancreatitis was diagnosed based on typically sonographic features of chronic calcific pancreatitis or other confirmatory imaging findings.

Descriptive statistics were used to summarize the data. The cross-table method with the chi-square test was applied to assess the association between the two groups. All analyses were conducted using SPSS software (version 20.0, Chicago, IL, USA). A *p*-value of less than 0.05 was defined as statistically significant.

## 3. Results

**Figure 1:**
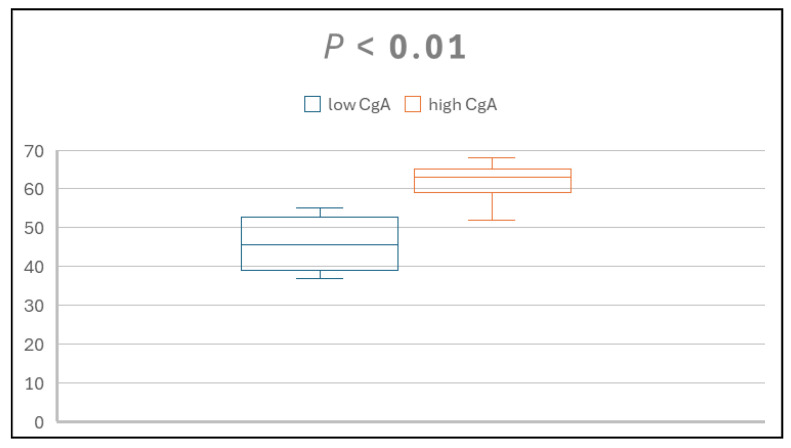
The comparison between age and serum CgA low and high groups. X axis: groups; Y axis: age.

A total of 15 patients diagnosed with dyspepsia participated in this study, 9 males and 6 females. Participants’ ages ranged from 37 to 68 years. Using a CgA cutoff value of 50 ng/mL, four patients were classified into the low group and eleven into the high group. Analysis indicated a positive correlation between age and serum CgA level, as illustrated in Figure 1.

Within the low CgA group, CgA values ranged from 29 to 35 ng/mL, and ages spanned 37 to 55 years. Diagnoses for these four individuals included one case each of duodenal ulcer, gallstone, functional dyspepsia, and concurrent duodenal ulcer and gallstone. *Helicobacter pylori* infection was present in both cases involving duodenal ulcers.

In the high CgA group, CgA concentrations ranged from 60 to 132 ng/mL, with ages ranging from 52 to 68 years. Diagnoses for these eleven participants included five cases of functional dyspepsia, two cases of gallstone, three cases of pancreatic fibrosis, and one case of alcoholic chronic calcific pancreatitis. Demographic information for the low and high CgA groups is provided in Table 1.

**Figure 2:**
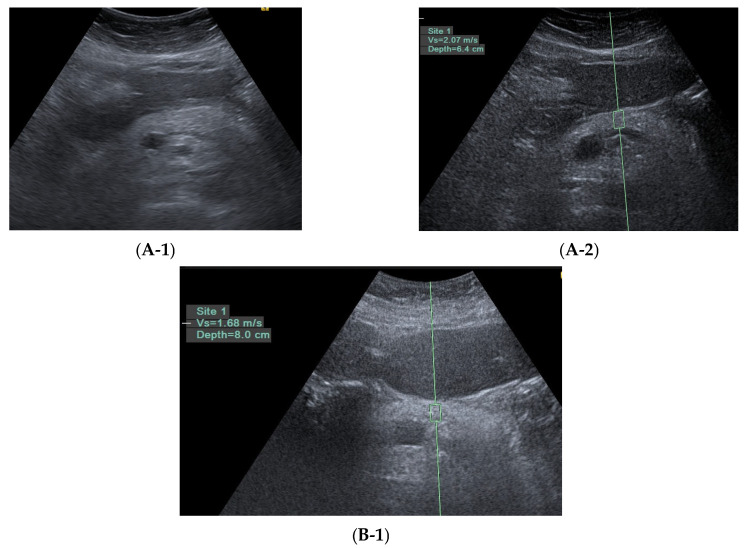

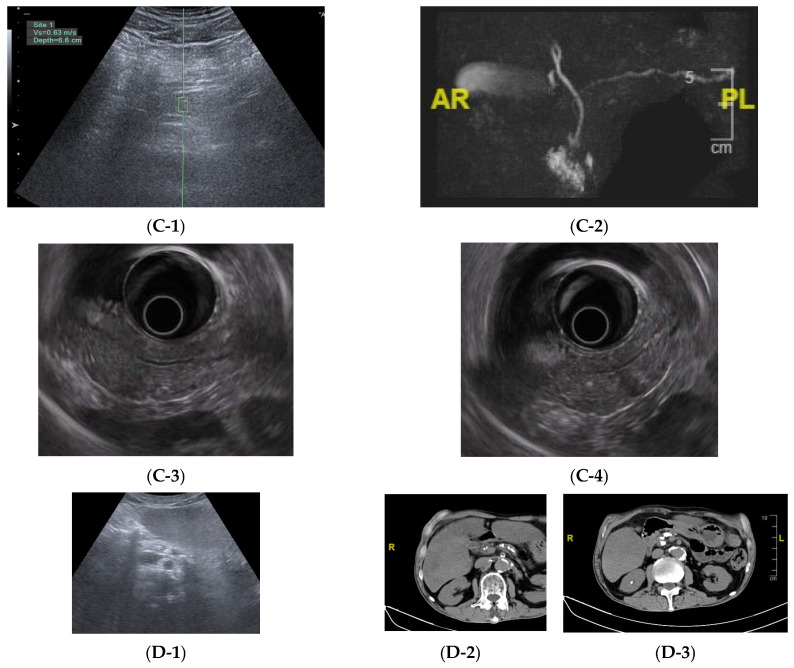
Image evidence of pancreatic fibrosis (*n* = 3) and chronic calcific pancreatitis (*n* = 1). Case 1: a 64-year-old female, abdomen sonography showed heterogenous pancreatic echotexture (**A-1**) and pancreatic acoustic radiation force impulse: 2.07 m/s (**A-2**). Case 2: a 52-year-old female, abdomen sonography showed pancreatic acoustic radiation force impulse: 1.68 m/s (**B-1**). Case 3: a 59-year-old man with normal pancreatic acoustic radiation force impulse (**C-1**) but serum CgA 121 ng/mL. The magnetic resonance image showed dilated pancreatic duct at tail part (**C-2**). Endoscopic ultrasonography showed pancreas fibrosis: main pancreatic duct hyperechoic edge, hyperechoic foci without acoustic shadow, dilated side branches, faint strandings (**C-3**,**C-4**); the diagnosis of pancreatic fibrosis is based on the Japan endoscopic ultrasound criteria for early chronic pancreatitis, 2019 edition [17]. A 68-year-old man with alcoholic chronic calcific pancreatitis. Typical trans-abdominal sonography (**D-1**) and computed tomography (**D-2**,**D-3**).

Of the three patients with pancreatic fibrosis, two were diagnosed using pancreatic acoustic radiation force impulse elastography, while one was identified through magnetic resonance imaging performed during surveillance for elevated serum CgA; no pancreatic neuroendocrine tumor was detected in this patient. However, evidence of pancreatic fibrosis was observed, including pancreatic duct dilatation at the tail region. This patient also underwent endoscopic ultrasound examination, which confirmed pancreatic fibrosis. Imaging findings from all three cases of pancreatic fibrosis and one case of chronic calcific pancreatitis are presented in Figure 2.

An analysis of duodenal EEC distribution patterns, as assessed via duodenal mucosa biopsy specimens, revealed that the low CgA group predominantly exhibited a peripheral and discrete pattern in 83.3% of cases (10/12). Conversely, a clustered pattern was mainly observed in the high CgA group, accounting for 84.8% of cases (28/33), a difference found to be statistically significant (*p* < 0.01). Examination of specific diseases showed that pancreatic fibrosis and chronic calcific pancreatitis were highly consistent with a clustered and more uniform pattern (91.7%, 11/12), whereas duodenal ulcers were predominantly fewer and displayed a discrete pattern (83.3%, 5/6). Mixed patterns, including both discrete and clustered types, were noted in gallstone and functional dyspepsia cases. A significant difference in serum CgA levels was identified between duodenal ulcer and pancreatic fibrosis/chronic pancreatitis groups (*p* < 0.01).

Further investigation into biopsy specimens from various duodenal locations within the same individuals indicated varying degrees of consistency. In the low CgA group, two cases exhibited complete concordance, while another two demonstrated partial consistency with only two specimens matching (two of three specimens). The high CgA group included six cases with entirely consistent results and five with partial consistency involving two specimens (two of three specimens). Associations between EEC distribution patterns, serum CgA levels, and disease subtypes are detailed in Table 2.

**Figure 3:**
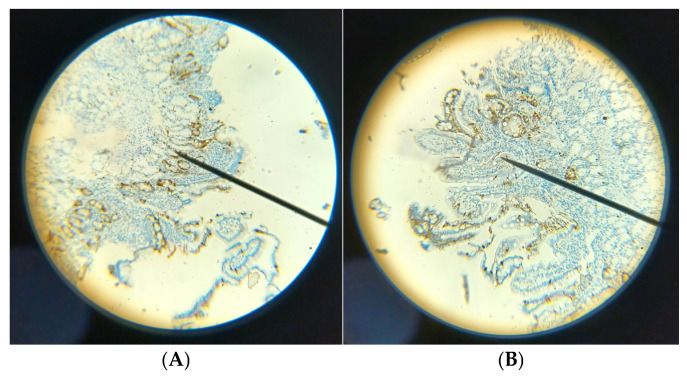
The EEC clustered and arranged as a belt-like pattern in functional dyspepsia (93%), (**A**). The EEC clustered and scattered more uniformly in pancreatic fibrosis and chronic pancreatitis (91%), (**B**). *p* Value < 0.01.

Within the high CgA group, five patients with functional dyspepsia presented with a clustered EEC pattern distinct from that seen in pancreatic fibrosis and chronic pancreatitis. Functional dyspepsia cases featured a belt-like clustering shape of EECs in 93.3% (14/15), while pancreatic fibrosis and chronic pancreatitis showed a more uniformly clustered type in 91.7% (11/12). Representative examples of these differing EEC distribution types for functional dyspepsia and pancreatic fibrosis are illustrated in Figure 3.

## 4. Discussion

The hypothesis that serum CgA levels are associated with various dyspeptic diseases and the distribution pattern of duodenal EECs formed the basis of this investigation. The rationale was that serum CgA levels exhibit a sufficiently broad range to encompass various non-malignant dyspeptic disorders and may also be correlated with aging [11,18,19]. This study demonstrated a relationship between serum CgA concentration and the spatial arrangement of duodenal EECs. In patients with low CgA levels (<50 ng/mL), EECs existed mostly as isolated, discrete cells groups. In contrast, individuals in the high CgA group (>50 ng/mL) displayed a centralized and clustered EEC distribution, suggestive of increased intercellular interactions [20]. Earlier studies suggest that small bowel EEC distribution is linked to pathophysiological functions [21]. Accordingly, it is plausible to infer that the clustering observed in patients with elevated CgA reflects an augmented degree of gut hormone interplay.

In the high CgA group, the typical diseases identified were chronic pancreatic fibrosis and chronic pancreatitis. Previous research has indicated a relationship between dyspepsia and pancreatic dysfunction in cases of both pancreatic fibrosis and chronic pancreatitis [12,22,23]. The clustered distribution of duodenal EECs observed in this group may be related to intercellular communication that supports pancreatic digestive function. Most specimens in the low CgA group showed peripheral, discrete EEC patterns, typical of duodenal ulcer disease. In duodenal ulcers, the upper gastrointestinal tract may require more rest for mucosal wound healing. Consequently, duodenal EECs would decrease gut hormone interactions, which may contribute to the observed peripheral and discrete pattern in this group [24]. This study finding supports the rationale of small bowel EECs undergoing dynamic change when the duodenum mucosa suffers injury [13].

Previous Japanese research has underscored the significant role of duodenal mucosal cells in the pathophysiology of functional dyspepsia and early chronic pancreatitis [25,26]. Among the various duodenal cells and gut hormones, glucagon-like peptide 1 (GLP-1)—a gut hormone involved in the regulation of gastric emptying and pancreatic function—has been postulated to contribute to the development of early chronic pancreatitis. Nonetheless, prior investigations have not demonstrated statistically significant correlations between GLP-1 expression and disease status. This lack of association may be attributable to the fact that GLP-1 is secreted by intestinal L-cells in response to food intake, resulting in diminished expression during fasting—a prerequisite before endoscopic duodenal biopsy. Furthermore, assessing only GLP-1-positive cells may not adequately reflect the complex interplay of gut hormones. Consequently, the present study focused on duodenal EECs, as they are primary progenitors for all gut hormone-producing cell types and may provide a comprehensive surrogate marker.

In this study, three cases were diagnosed with pancreatic fibrosis. All three underwent pancreatic elastography surveillance, but only two met the criterion of shear wave velocity exceeding 1.4 m/s. The third case demonstrated a pancreatic shear wave velocity below 1.4 m/s, while serum CgA was above the upper normal limit at 121 ng/mL. For pancreatic neuroendocrine tumor surveillance, magnetic resonance cholangiopancreatography was performed for the third case. No neuroendocrine tumor was detected; however, dilatation of the main pancreatic duct in the tail region was observed. Considering the ductal dilatation and a family history of pancreatic adenocarcinoma, pancreatic fibrosis was diagnosed in this case. Then, pancreatic fibrosis was confirmed through endoscopic ultrasound examination in the third case.

In this series of three patients with pancreatic fibrosis, transabdominal shear wave elastography demonstrated a sensitivity of 66.67% (2/3). According to the literature, the sensitivity of point shear wave velocity for typical late-stage chronic pancreatitis is approximately 75–91% when using a cutoff value of 2.09 m/s [8]. Transabdominal sonographic measurement of pancreatic shear wave velocity may be influenced by adjacent organs, bowel gas, and patient cooperation. Therefore, further investigation into additional methods to enhance the diagnostic sensitivity for pancreatic fibrosis is warranted.

Serum CgA has demonstrated potential as a diagnostic biomarker for chronic pancreatitis and pancreatic fibrosis [27]. Chronic pancreatitis, whether in early or late stages, is typically associated with fibrosis. Accordingly, it is reasonable to hypothesize that during the fibrotic progression of the pancreas, CgA-positive pancreatic cells may secrete CgA into the bloodstream [27]. A threshold value of 50 ng/dL is suggested, given that all individuals with pancreatic fibrosis or chronic pancreatitis exhibited serum CgA concentrations greater than 60 ng/dL in this study. These findings align with earlier studies reporting an increased proportion of CgA-positive cells in chronic pancreatitis, supporting the observed elevation in serum CgA levels. Furthermore, this investigation identified a positive correlation between serum CgA concentrations and patient age (*p* value < 0.05), which may indicate an age-related compensatory mechanism. As organ function diminishes over time, augmented CgA production could contribute to the maintenance of physiological homeostasis. This age- and serum level-positive association was consistent with other research results [28].

This study presents several limitations. First, it does not include a quantitative assessment of CgA-positive cells in the duodenal mucosa across different diseases. Second, it does not identify the proportion of circulating CgA derived specifically from the duodenal mucosa, considering CgA is secreted by various organs. Third, possible intra- and inter-observer variability in evaluating EEC patterns may result in bias. Fourth, this research is a case series with a limited number of cases.

In clinical practice, dyspepsia, peptic ulcer disease, and gallstones are generally straightforward to diagnose. However, diagnosing pancreatic fibrosis can be challenging. In addition to imaging techniques, measurement of serology CgA above 50 ng/mL and targeted duodenal mucosa biopsy (with at least three specimens) for analysis of EEC patterns may serve as supplementary approaches for diagnosis.

In summary, serum CgA levels demonstrate a significant correlation with patterns of EEC distribution in the duodenal mucosa. A threshold of 50 ng/dL effectively differentiates between centralized and clustered EECs—typically observed in pancreatic fibrosis and chronic pancreatitis—and peripheral and discrete EECs, which are more frequently associated with duodenal ulcers. Distinct EEC distribution patterns were observed in pancreatic fibrosis compared to functional dyspepsia when serum CgA > 50 ng/mL. These results underscore the potential value of duodenal mucosal biopsy for EEC distribution analysis and the potential use of serum CgA as a biomarker for diagnosing chronic pancreatitis and pancreatic fibrosis.

## Figures and Tables

**Table 1:** Demography of low and high serum CgA groups.

	CgA < 50 (ng/mL)	CgA ≥ 50 (ng/mL)	*p* Value
Serum CgA range	29~35	60~132	<0.01
Total no. of cases	N = 4	N = 11	#
Age (years)	37~55	52~68	<0.01
Gender (male/female)	2/2	7/4	#
Functional dyspepsia	1	5	0.46
Duodenal ulcers	2	0	0.05
Gallstone	2	2	0.27
Pancreatic fibrosis	0	3	0.36
Chronic pancreatitis	0	1	0.73
Diabetes mellitus	0	2	#
*H. pylori* infection (pathology)	2	1	#
Renal insufficiency	0	0	#
Atrophic gastritis and intestinal metaplasia (pathology)	1	4	#
Proton pump inhibitor use	1	1	#

# Statistical test was not performed.

**Table 2:** Associations between serum CgA with duodenal EECs distribution pattern or specific disease.

Classification		*p* Value
CgA < 50 ng/mL group (biopsy specimen *n* = 12)	CgA > 50 ng/mL group (biopsy specimen *n* = 33)	
Discrete, fewer 83.3% (10/12)	Centralized, clustered 84.8% (28/33)	<0.01
Consistency of the same individual	Consistency of the same individual	
3 of 3 specimens: 2 cases	3 of 3 specimens: 6 cases	
2 of 3 specimens: 2 cases	2 of 3 specimens: 5 cases	
CgA comparison between specific diseases		
Duodenal ulcer (*n* = 2)	Pancreatic fibrosis, and chronic pancreatitis (*n* = 4)	
29, 35	62, 78, 119, 121	<0.01
EEC distribution pattern 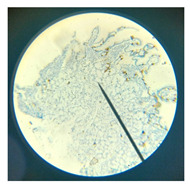	EEC distribution pattern 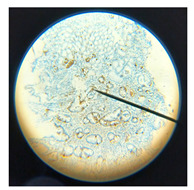	

## Data Availability

The raw data supporting the conclusions of this article will be made available by the authors on request.
